# Navigation of Silver/Carbon Nanoantennas in Organic Fluids Explored by a Two-Wave Mixing

**DOI:** 10.3390/nano10091886

**Published:** 2020-09-21

**Authors:** Geselle García-Beltrán, Cecilia Mercado-Zúñiga, Christopher René Torres-SanMiguel, Martín Trejo-Valdez, Isaela Villalpando, Carlos Torres-Torres

**Affiliations:** 1 Sección de Estudios de Posgrado e Investigación, Escuela Superior de Ingeniería Mecánica y Eléctrica Unidad Zacatenco, Instituto Politécnico Nacional, Ciudad de México 07738, Mexico; ggarciab1102@alumno.ipn.mx (G.G.-B.); ctorress@ipn.mx (C.R.T.-S.); 2 Departamento de Ingeniería de Materiales, Tecnológico de Estudios Superiores de Coacalco, Cabecera Municipal 55700, Mexico; cecilia@tesco.edu.mx; 3 Escuela Superior de Ingeniería Química e Industrias Extractivas, Instituto Politécnico Nacional, Ciudad de México 07738, Mexico; mtrejov@ipn.mx; 4 Centro de Investigación para los Recursos Naturales, Salaices 33941, Mexico; i.villalpando@cirena.org

**Keywords:** nonlinear optics, two-wave mixing, silver nanoparticles, carbon nanostructures, diffusivity, nanoantenas

## Abstract

Within this work are analyzed third-order nonlinear optical properties with a potential influence on the dynamic mechanics exhibited by metal/carbon nanofluids. The nanofluids were integrated by multiwall carbon nanotubes decorated with Ag nanoparticles suspended in ethanol or in acetone. Optical third-order nonlinearities were experimentally explored by vectorial two-wave mixing experiments with a Nd-YAG laser system emitting nanosecond pulses at a 532 nm wavelength. An optically induced birefringence in the metal/organic samples seems to be responsible for a significant modification in density and compressibility modulus in the nanosystems. The measured nonlinear refractive index was associated with a thermal process together with changes in density, compressibility modulus and speed of sound in the samples. Nanofluid diffusivity was studied to characterize the dynamic concentration gradients related to the precipitation of nanostructures in the liquid solutions. The evolution of the nanoparticle density suspended in the nanofluids was considered as a temporal-resolved probabilistic system. It is stated that the incorporation of Ag nanoparticles in carbon nanotubes produces strong mechanical changes in carbon-based nanofluids. According to numerical simulations and optical evaluations, immediate applications for developing dynamic nanoantennas optical logic gates and quantum-controlled metal/carbon systems can be contemplated.

## 1. Introduction

Photoinduced functions tailored by nonlinear optics and materials science correspond to an attractive field of research regarding the possibility to design ultrafast and low-dimensional applications. Nanostructured materials have emerged as a new generation of advanced materials with strong sensitivity to shape, size and distribution that determine their physical and chemical performance. In this direction, the inclusion of hybrid nanostructures in organic nanofluids has been related to an automatic enhancement in their third-order nonlinear optical effects.

Carbon nanotubes (CNTs) exhibit remarkable optical [1] and mechanical properties as the apparent density [2] and a notable Young modulus [3]. The peculiar morphology of CNTs consists of rolled-up structures of pure carbon with nanometric diameters and lengths of many microns. The structure of CNTs allows their use as a support material for the dispersion and stabilization of metal and semiconductor nanoparticles (NPs) [4]. The decoration of CNTs allows for integrating the properties of metallic NPs in carbon structures, resulting in unique optical and mechanical properties [5]. In addition, Ag NPs stand out from other materials with plasmonic response because of their optical selectivity. Ag also provides a strong enhancement in plasmonic and absorption effects useful for optical processes [6]. Plasmonic interactions of Ag NPs could find applications as optical sensors or broadside nanoantenas [7]. Metallic NPs can act as optical antennae, due to their reception range and emission of optical radiation. The incorporation of metallic NPs on nanofluids can produce sharp-selective optical interactions; in particular, Ag NPs have a large third-order nonlinear optical response at wavelengths close to the absorption band of their surface plasmon resonance [8]. Ag colloidal solutions in organic liquids exhibit a large thermal-induced nonlinear refractive index [9]. Photoinduced energy transfer is a consequence of the high thermo-optic coefficient of organic fluids as ethanol [10]. In addition, the advantages of the functionalized CNTs stand out in the changes in physical properties that imply improvements in solubility and dispersion of carbon nanofluids [11]. Multiwall CNTs (MWCNTs) in nanofluids originate in an important change in effective thermal conductivity and viscosity [12]; however, it is important to consider the characteristics of the solvent [13]. Remarkable properties such as high thermal conductivity and high energy vaporization for ethanol and acetone are present in carbon nanofluids [14]. Many organic fluids have been useful in nanofluid applications due to their outstanding magnetic [15], thermal [16], and optical phenomena [17]. Carbon-based nanomaterials [18], metal NPs [19] and carbon-based nanofluids [20] exhibit a nonlinear optical response over a large wavelength range. In addition, the incorporation of plasmonic NPs in carbon nanostructures could be responsible for modifying optical nonlinearities induced by intense optical waves in nanofluids. Therefore, carbon nanofluids based on ethanol or acetone have high sensitivity to optical and mechanical effects [21]. Nanostructures with high nonlinear refractive index are of interest due their fascinating applications for developing all-optical switching devices. Progress in optical research has allowed the design of quantum systems based on Kerr nonlinearities that produce phase-changes dependent on optical irradiance [22]. Moreover, quantum optics has demonstrated several methods to generate optical nonlinearities in photons for developing optical gates [23], and the influence of optical waves on dynamics in nanofluids extends the possibility for exploring different remote or low-dimensional atmospheres.

The identification of optical nonlinearities exhibited by nanostructures in precipitation through nanofluids can be considered for teledetection or revelation of physical phenomena correlated with results obtained by laser scanning. Particular nonlinear optical parameters exhibited by nanofluids can present a strong influence in the density of their suspended NPs. The non-uniform speed of the nanoantenas can also be employed for the smart detection and identification of biofluids with biological conditions sensitive to the dynamics of the NPs [24].

With these motivations, the impact of this research mainly corresponds to the study of plasmonic nanoantenas with probabilistic logic functions controlled by dynamic nonlinear optical properties. Silver decorated MWCNTs suspended in organic nanofluids were evaluated. We analyzed third-order nonlinear optical phenomena and probabilistic effects based on diffusivity of the samples and the optical monitoring of concentration gradient associated with the particles suspended in the studied nanofluids. This work was devoted to further investigating potential probabilistic gate functions based on mechanical and Kerr nonlinearities exhibited by nanofluids. Analogic signals provided by nonlinear sensors are not preferable in respect to digital sensors in regard to the fact that observational errors usually cause excessive sensitivity to variations in environmental conditions. In this respect, we propose a logic gate system to identify the information provided by the nanostructures studied. A digital logical operation is defined in this work in terms of nonlinear optical signals in propagation through the nanoantenas in dynamic precipitation along the nanofluids. We consider that our strategy opens up an alternative for collecting information by nonlinear sensors and revelation of the evolution of nanofluids properties by probabilistic signal processing functions.

It is remarkable that silver decorated CNTs in an organic liquid solution can provide a hybrid nanofluid with optical nonlinearities improved by plasmonic phenomena. This work highlights that the third-order nonlinear optical behavior of metal/carbon nanofluids can be considered for developing electromagnetically controlled functions and dynamic nanoantennas.

## 2. Materials and Methods

### 2.1. Sample Preparation and Morphology Characterization

The MWCNTs growing process was carried out by the spray pyrolysis method. This growing method consists of setting a quartz tube inside a cylindrical oven at 850 °C. The quartz tube was fed with a toluene and ferrocene solution. Toluene molecules break down into carbon atoms and they are hexagonally arranged in the form of MWCNTs due to the presence of iron NPs derived from the decomposition of ferrocene [25]. Subsequently, MWCNTs were functionalized in 3:1 *v/v* mixture of 30 mL of 95–97% sulfuric and 10 mL of 65% nitric acid under sonication at 42 kHz for 15 min at room temperature. Functionalized MWCNTs were repeatedly washed in distilled water, centrifuged and dried in vacuum. Functionalization of MWCNTs with HNO_3_/H_2_SO_4_ solution resulted in the formation of a surface associated with carbonyl, carboxyl, and hydroxyl functional groups. The metal decoration process of MWCNTs with Ag NPs was carried out by a chemical vapor deposition method. For each milligram of MWCNTs, 5.3 mg of Ag, 25 mL of dimethyl sulfoxide (DMSO) and 0.85 g of AgNO_3_ were used. The samples were exposed to ultrasound for 10 min. The mixture was in continuous agitation at 120 RPM with a constant temperature of 60 °C for 30 min. After agitation, a solution was obtained due to the dissociation of the AgNO_3_ precursor agent with the reducing agent DMSO. The samples were filtered and rinsed with acetone to remove the impurities. A filtering process was carried out in a vacuum, and then the samples were placed on a glass platform to be dried. The washing and filtering steps were repeated 4 times. Finally, to completely dry the samples, the glass platform was placed in a muffle at 200 °C for 30 min.

Previous results in comparative MWCNTs indicate that the influence of the inclusion of single-wall CNTs (SWCNTs) or noble metal NPs in MWCNTs importantly modulate their collective nonlinear optical response [26]. SWCNTs can present opposite nonlinear optical effects in respect to MWCNTs and Ag NPs can switch the physical mechanism responsible for multiphotonic absorption [27]. Hierarchical nanostructures with different concentration of metal NPs revealed that the coating of the tubes can be completed with a volume fraction of NPs in respect to the tubes of about 1:3 [28]. With these considerations, we designed our sample with the incorporation Ag NPs integrated in the MWCNTs as Ag-MWCNT samples.

Ag-MWCNTs were weighted to prepare the nanofluid samples measured in this work. Then, 8.2 mg of Ag-MWCNTs were suspended in an acetone solution with a volume of 2 mL. Comparatively, 8.2 mg of Ag-MWCNTs were suspended in an ethanol solution with a volume of 2 mL. Different concentrations of the nanostructures in the selected organic samples were analyzed. Here are described the conditions of the most representative results with heuristically chosen concentrations to better observe the optical nonlinearities without a strong depletion of the optical transmittance and a high signal to noise ratio.

In order to analyze the metal decoration and chemical composition in MWCNTs, field-emission scanning electron microscopy with energy-dispersive X-ray (SEM and EDX; JEOL JSM-6701F) studies were undertaken. High-resolution transmission electron microscopy (TEM) studies were carried out to confirm the multiwall nature of the CNTs studied.

### 2.2. Third-Order Optical Nonlinearities Explored by a Two-Wave Mixing

**Figure 1:**
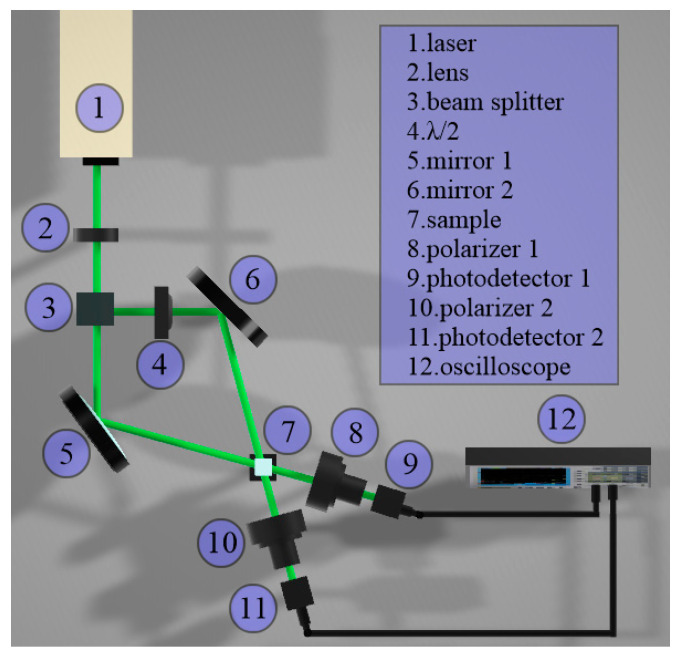
Scheme of the experimental setup of the two-wave mixing (TWM).

A two-wave mixing (TWM) technique was conducted in order to evaluate the third-order nonlinear optical response of the nanofluid samples. The schematic experimental setup for the superposition of the two coherent and polarized beams is illustrated in Figure 1. A Nd-YAG laser system (Continuum model SL II-10) with 4 ns of pulse duration and wavelength at 532 nm was used as an optical source. The pump and probe beams with an optical irradiance relation of 1:10 were focused in the nanofluid sample S contained in a quartz cuvette with 1 mm thickness. The spot size of the focused beams was approximately 1 mm. The propagation vectors of the beams make a geometrical angle of 30° due to the beam splitter and the mirrors. A half-wave plate, λ/2, modifies the angle of the polarization plane of the incident pump beam. A PIN photodetector was used to measure the orthogonal polarization component of the incident probe beam transmitted through a polarizer by the influence of the pump beam. The maximum total optical irradiance of the probe beam in the sample was 2 MW/cm^2^. The high-irradiance of the pump beams promotes the modification of the polarization state of the probe beam. Likewise, the modification of the polarization state of the beams results in changes in the transmittance of the probe beam in the TWM system.

The electric fields in propagation through the samples can be described by considering the wave equation [29] as described in the supplementary material.

According to Equation (S3), the intensity-dependent refractive index can be significant for high-irradiance effects induced in a nonlinear material. For nanosecond pulses irradiating MWCNTs, the most likely physical mechanism responsible for the nonlinear refractive index, *n*_2_, is the photothermal effect [30], and this third-order optical nonlinearity excited by an important photoinduced energy transfer can also be responsible for a local modulation in density in the irradiated sample [26].

#### Photoinduced Mechanical Response

Considering the optical Kerr effect (OKE) represented by Equation (S3), the density of a nonlinear optical medium can be modified. Then, from Equations (S3) and (S4), the change in mass density, Δ*ρ_m_*, coming from the OKE can be estimated by,

(1) $$\Delta \rho_{m}=\left[\frac{{\left(n_{0}+n_{2}I\right)}^{2}-1}{{\left(n_{0}+n_{2}I\right)}^{2}+2}-\frac{n_{0}^{2}-1}{n_{0}^{2}+2}\right]\left[\frac{3M}{4\pi N_{A}\alpha }\right]$$

where *n*_0_ is the weak-field refractive index, *n*_2_ in the nonlinear refractive index, *I* refers to the intensity of the optical field, *α* is the polarizability, *N_A_* is the Avogadro’s number and *M* is the molecular weight of the chemical element.

In Equation (1), it is implicit that a change in irradiance generates a change in the refractive index. Explicitly, Equation (1) also describes that a change in the density of mass can be obtained as a function of irradiance in the sample.

In addition, the compressibility modulus, Δ*Y*, can be estimated as a function of the speed of sound through the medium, *v_a_*, and the change in density with dependence on the OKE:

(2) $$\Delta Y=v_{a}{}^{2}\left[\frac{{\left(n_{0}+n_{2}I\right)}^{2}-1}{{\left(n_{0}+n_{2}I\right)}^{2}+2}-\frac{n_{0}^{2}-1}{n_{0}^{2}+2}\right]\left[\frac{3M}{4\pi N_{A}\alpha }\right]$$

### 2.3. Effects of Difusivity in Plasmonic Nanoantennas

The second Fick law is a mathematical description of the evolution exhibited by a diffusion effect by a partial differential equation. This law is described by [31]:

(3) $$\frac{\partial C}{\partial t}=D\frac{\partial^{2}C}{\partial x^{2}}$$

where *C* is the concentration of the solute in the solvent, *D* is the diffusion coefficient, and *x* is the position of the particle. The solution of the differential equation corresponds to the probability at the coordinates of the particles *x* of the medium at each time *t* [32].

(4) $$p(x,t)=\frac{1}{\sqrt{4\pi Dt}}\exp\left(\frac{-x^{2}}{4Dt}\right)$$

In order to determine the diffusivity coefficient value *D_S1−S2_*, where *S*1 is the solute, *S*2 is the solvent, the Wilke–Chang relation can be used [33]:

(5) $$D_{S1-S2}=\delta \left[\frac{\varphi \sqrt{\vartheta M_{S2}}}{{ϒ}_{S2}{\left(\upsilon_{S1}\right)}^{0.6}}\right]$$

where *δ* is a constant equivalent to 1.17 × 10^−16^, ϑ is the association parameter of the fluid, *M_S_*_2_ is the molecular weight of the solvent, ϒ*_S_*_2_ is the viscosity of the solvent, and υ*_S_*_1_ is the atomic volume of the solute and *φ* is the temperature.

The particles of a nanofluid are in constant fluctuation. That means the absorption of a sample is modified as a function of the time related to the stability of the sample. A numerical fitting for describing the temporal dynamics of a nanofluid can be computed by considering:

(6) $$y=ae^{-t/\tau }+b$$

where τ is the value of the independent variable time, *y* represents the absorbance, a is a constant and *b* is the point where the straight line cuts the vertical axis.

### 2.4. Probabilistic Signals Exhibited by Plasmonic Nanoantenas

In contrast to the classic digital recording of binary bits where only the values 0 and 1 can be present, the probabilistic bits refer to an overlap of values between 0 and 1. A probabilistic bit system is a two-dimensional complex vector space, and the state of a probabilistic bit, Ψ, can be represented by a complex vector in that space as follows:

(7) Ψ=Γ|0〉+Λ|1〉

The configurations of the probabilistic bits increase infinitely due to coefficients that can be negative and with complex numbers. The probabilistic bits are characterized by the complex numbers Γ and Λ, which correspond to the probability of measuring a value of 0 and 1. Likewise, the notation ket | 〉 means input and the notation 〈 |, means output. Since these coefficients can be imaginary, they cannot simply be interpreted as probabilities of their associated results. Then, the probability of measuring each state is given by [34],

(8) $${\left|\Gamma \right|}^{2}+{\left|\Lambda \right|}^{2}=1$$

The quantum logic gates can be represented by reversible operations between the quantum elements. A three probabilistic bits gate can be described as a controlled gate with inputs S1–3 and outputs O1–3. In this case, S1 corresponds to O1, S2 corresponds to S2 and S3 corresponds to O3. However, O3 changes its value if S1 and S2 are 1. It can be interpreted as a logic gate with 2 control probabilistic bits identified as S1 and S2. The truth table is shown in Table 1.

This gate is usually employed for simulating irreversible classic gates. Among its particularities, it can be mentioned that this gate is one of the most important classic gates, the NAND gate.

Different architectures based on carbon nanomaterials have demonstrated exceptional optical nonlinearities that can be tailored by their structure and morphology [35]. This particular physical empowering related to size, shape and chirality has been employed in MWCNT systems to design unusual metal/carbon nanohybrids assisted by decoration processes including plasmonic NPs [36]. It is worth mentioning that silver NPs present a sharp selective absorption band associated to their characteristic surface plasmon resonance close to 400 nm, while MWCNTs exhibit their characteristic resonance around 270 nm [27]. The integration of a double resonance characteristic by Ag NPs in MWCNTs seems to be attractive with advantages for visible laser driven logic gate functions and single-photon devices in the UV region of the electromagnetic spectrum. Optical nonlinearities exhibited by nanostructures in precipitation through nanofluids can be considered for remote sensing or laser scanning as a function of their dynamics. The use of MWCNTs decorated with Ag NPs has been chosen by considering that metallic NPs can act as nanoantenas with an enhanced reception and emission of optical radiation in nonlinear devices. The operation of nanoantenas is based on capturing an electromagnetic wave of a specific wavelength by an energy transfer. In our case, this nanoantenna response is considered to be plasmonic and achieved by the collective excitation of the electrons in the metal as a consequence of the strong interaction with the incident light. To improve metal light emitters, there is a coherent oscillation of the electrons confined to the surface of particular NPs. In Ag NPs, their absorption peak is maximum in the visible region of the electromagnetic spectrum. This response depends on factors such as the concentration of the NPs, spatial distribution, morphology and surrounding properties. The morphology of the studied nanostructures and their influence on optical properties with ethanol and acetone as background media was considered. The results corresponding to the absorption spectrum show that Ag NPs are attractive for applications in the visible range and close to UV.

## 3. Results

**Figure 2:**
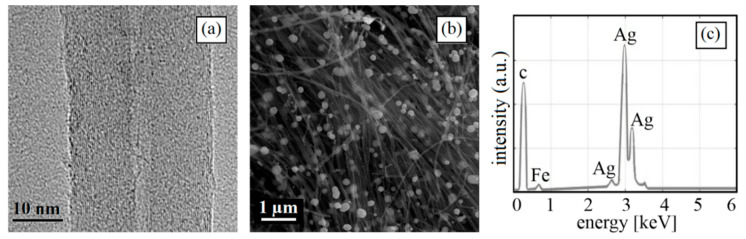
(**a**) A representative TEM micrograph of the studied carbon nanotubes (CNTs) confirming their multiwall nature. (**b**) SEM micrograph of representative regions of the samples. (**c**) Results of statistical EDX analysis for the studied silver decorated multiwall CNTs (Ag-MWCNTs).

Figure 2 shows the characterization of MWCNTs in order to reveal the multiwall nature, functionalization, and decoration of the CNTs. Figure 2a shows a high-resolution TEM image of a representative section of MWCNT growth. Bright-field mode was chosen to observe in detail the cross-section multiwall nature of CNTs. TEM micrograph provides clear evidence of the formation of multilayer nanotubes during the toluene–ferrocene decomposition reaction, which is several microns in length. In addition, the TEM studies determined that the internal diameters are approximately 4 nm ± 5% and the external diameters are up to around 28 nm ± 2%. Moreover, the intermolecular distance between the carbon layers was 0.134 nm.

SEM micrographs allowed the observation of the homogeneity of metal NPs incorporated to the MWCNT samples. Figure 2b shows the Ag NPs represented by quasi-circular white points in the extremes of MWCNTs. Ag NPs have an average size of approximately 60 nm ± 5%. Metal particles represent 30% of weight of MWCNTs. Correspondingly, from the image, it is possible to deduce that MWCNTs have a minimum length of 10 µm approximately.

Figure 2c depicts the elemental analysis of MWCNTs by the EDX technique. This analysis highlights the presence of carbon corresponding to MWCNT structure and iron appropriate of the synthesis process. There is also a notable presence of Ag NPs in the composition of the samples due to decoration process.

**Figure 3:**
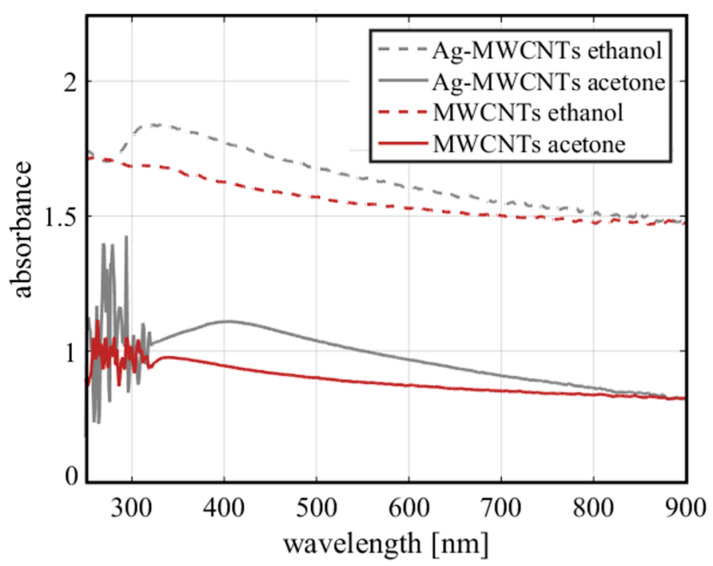
Typical UV-VIS absorption spectra of the studied nanofluids.

The UV-VIS absorption spectra of the samples prepared in ethanol and acetone with Ag-MWCNTs respectively are plotted in Figure 3.

From the spectra shown in Figure 3, it is possible to observe an emerging band corresponding to the excitation of the surface plasmon resonance of the Ag NPs near to 400 nm. However, an interesting point to highlight it is the influence of the geometry of the Ag NPs and the solvent in which they are suspended on the resonance spectral location [37]. The absorption band of pure acetone is located around 250 nm [38]. In the nanofluid spectrum shown in Figure 3, related to acetone, there is a high noise caused by phenomena related to scattering. The absorption bands for ethanol should be close to 340 nm. The peak in the absorption band associated with the π-π bond resonance exhibited by MWCNTs is located in the ultraviolet region, near to 270 nm. Nevertheless, the absorption peaks of the MWCNTs can move due to the polarity of the solvent [39]. On the other hand, the presence of MWCNTs agglomeration influences the absorption spectrum of the samples, such that, from the UV-VIS spectra, it can be identified that the sample in the acetone suspension has a lower absorption than the ethanol sample. So, from the previous points and from the experimental observations, we can conclude that MWCNTs present more agglomeration in ethanol suspensions than in acetone.

**Figure 4:**
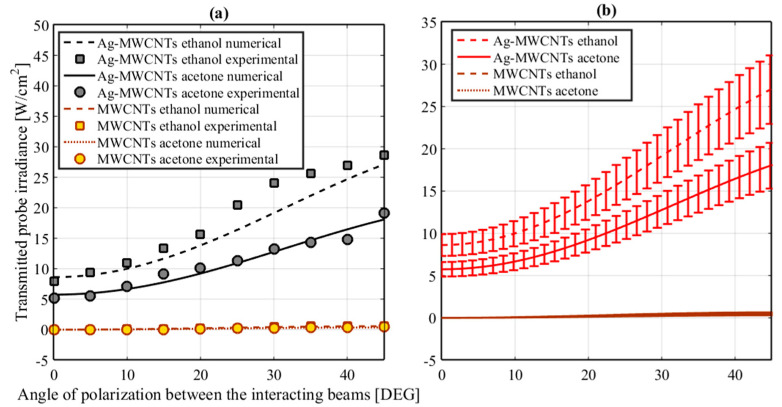
(**a**) Transmitted probe irradiance as a function of the angle between the planes of polarization of the incident beams in the nanofluids tested in the vectorial TWM. (**b**) Experimental error bars for (**a**).

To further investigate the possibility to modulate the dynamic response of the probabilistic logic gate, no changes in nonlinear optical absorption were observed through monitoring single-beam transmittance as a function of irradiance. However, we analyzed the OKE of the samples by our TWM experiment described by Figure 1. The calibration of the system was performed by using a CS_2_ sample. The TWM technique presents the potential to evaluate an induced birefringence promoted by the superposition of optical waves. From Figure 4, it is possible to observe experimental evidence of Kerr nonlinearities exhibited by Ag-MWCNT samples and a numerical fitting based on Equation (S1). The degenerated TWM method in a non-collinear pump-probe configuration was selected for measuring the nonlinear refractive index regarding the potential of this technique for identifying the physical mechanisms responsible for the optical Kerr effect [40]. Our analysis of Equation (S2) in Equation (S1) reveals that the fitting of numerical data is consistent with $X_{1212}^{\left(3\right)}$ = 0, which corresponds to the isotropic nonlinear refractive index [29]. The expected thermal effect induced by nanosecond pulses in MWCNTs [30] matches our results. The error bar corresponds to about ±15%. A strong magnitude in n_2_ = 6.1 × 10^−12^ cm^2^/W was obtained for the Ag-MWCNTs in ethanol and n_2_ = 5.6 × 10^−12^ cm^2^/W for the Ag-MWCNTs in acetone.

The third-order optical susceptibility of pure ethanol and pure acetone is omitted in Figure 4. This consideration is valid because the nonlinear optical responses of ethanol and acetone [29] are lower than the nonlinear results for the samples measured in this work, and they present a nonlinearity at least three orders of magnitude lower.

In our TWM experiment, pump and probe beams had incident linear polarizations, and their superposition in the sample produces a modulation in irradiance featuring an interference fringe pattern. The variation in irradiance along the sample automatically causes a modulation in nonlinear birefringence as a function of the change in refractive index given by Δn = *n*_2_I, with *n*_2_ being the nonlinear refractive index and *I* the optical irradiance profile. Initially, the incident polarization of the probe beam is fixed but sensitive to the nonlinear birefringence induced by the pump beam. The polarization of the pump beam was rotated by a half-wave plate during the experiment in order to explore the vectorial nature of the nonlinear birefringence induced during the interaction of the beams. An analyzer with its transmission axis orthogonal to the incident polarization of the probe beam monitored the evolution of the polarization of the probe beam after passing the sample. The transmitted probe irradiance is directly related to the characteristic third-order optical nonlinearities of induced birefringence and multiphotonic absorption exhibited by the studied samples in the TWM [29].

The nonlinear optical response of nanofluids can be used for the harmonic generation of optical waves through coherent optical mixing. In contrast with nanosecond optical effects, ultrashort pulses can be suitable for observing ultrafast electronic nonlinearities, but the modeling of thermal mechanisms that can be induced by picosecond and femtosecond pulses, which is more complex for describing photothermal energy transfer. Nanosecond pulses involve enough time for thermal transport while the photoinduced effects correspond to a collective response exhibited by nanofluids deposited in thicker samples [41]. The potential to excite stronger nonlinear optical absorption effects closer to the absorption band of the surface plasmon resonance of the Ag NPs can be expected by using the third-harmonic of our Nd:YAG laser system [42]; but this consideration is also correlated with a potential inhibition in the nonlinear optical refraction effects according to Kramers-Kroning relations.

Figure 4b corresponds to the error bar of the measurements associated with the transmitted probe irradiance as a function of the relative angle between the plane of polarization of the incident beams in the samples. These bars exhibit an error of approximately ±15%. From the absence of an overlapping in the experimental data for the Ag-MWCNTs in acetone and ethanol and the MWCNTs can be guaranteed the clear signature of the different studied samples in this TWM experiment.

The inherent morphology, structure and geometric properties exhibited by nanostructures are responsible for the outstanding changes in their optical, electrical and thermal behavior. In this regard, the incorporation of Ag NPs in MWCNTs in film form can promote the switching of multi-photonic effects that is topic of this research in nanofluids, as comparatively has been demonstrated in nonlinear optical absorption processes measured by single-beam techniques [27]. The uniform and homogeneous distribution of the metallic decoration as well as the multi-wall nature of the tubes were guaranteed and analyzed in this work by SEM, TEM and X-ray studies.

Furthermore, the incorporation of Ag NPs in carbon nanostructures seems to cause an enhancement in the nonlinear optical properties in comparison with carbon nanostructures without decorations [27]. The enhancement in the third-order nonlinear optical response can be attributed to surface plasmon absorption effects of Ag NPs [43]. A nonlinear absorption coefficient and nonlinear refractive index are strongly dependent on the NP concentration in the solutions and the duration of pulses [44]. In addition, a fast photoresponse is characteristic in CNT-decorated samples [45].

**Figure 5:**
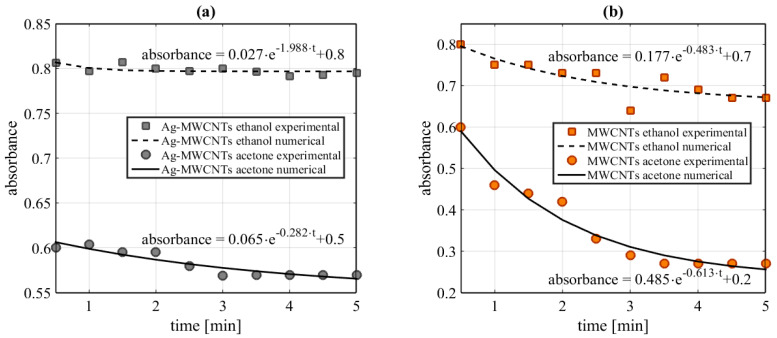
Time dependence of UV-VIS absorbance at 532 nm. (**a**) Ag-MWCNT samples, (**b**) MWCNT samples.

In order to measure the stability of nanofluids, the optical absorption at 532 nm of nanofluids was monitored after a sonication process in the samples. The UV-VIS spectra were evaluated in a range of 0 to 5 min, in order to demonstrate the stability of the samples. Figure 5 shows the dependence on absorbance in the time from the samples. The numerical fitting for the experimental measurements was obtained by Equation (6). It is possible to observe the parameters a, τ and b, related to the behavior of the numerical fitting for each sample.

The stability of nanofluids is strongly dependent on the solution but also changes with the incorporation of the NPs in the carbon nanostructures [46]. It has also been demonstrated that the stability times of Ag NPs nanofluids are longer than others [47]. It is worth mentioning that the time-dependence stability of nanofluids strongly depends on the diffusion of the suspended NPs and the organic solvents.

**Figure 6:**
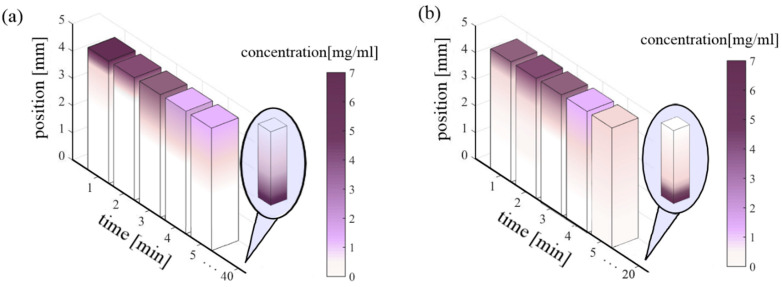
(**a**) Diffusivity of carbon nanostructures in ethanol. (**b**) Diffusivity of carbon structures in acetone.

The spatial distribution of molecules in a nanofluid is not homogeneous; this implies that there is a concentration gradient between two points in the medium. The diffusion phenomenon describes the concentration gradients with respect to time in the fluid. The concentration at the position of the particles in the medium at each instant of time obtained from Equation (4) is represented in Figure 6. The diffusion coefficient approximation was calculated from Equation (5) with Ψ_Ethanol_ = 1.5, Ψ_Acetone_ = 1, M_Ethanol_ = 46.07 g/mol, M_Acetone_ = 58.08 g/mol, ϒ_Ethanol_ = 1.074 mPa·s, ϒ_Ethanol_ = 0.32 mPa·s and ν_Carbon_ = 4.58 cm^3^/mol. The diffusion coefficient for carbon-acetone is 3 × 10^−13^ cm^2^/s and that for carbon-ethanol is 9 × 10^−14^ cm^2^/s. Time-dependence stability of the Ag-MWCNTs nanofluids strongly depends on the diffusion coefficient between the CNTs suspended and the ethanol or acetone solvents. The diffusion coefficient indicates an inverse relation in respect to the stability of the fluid. In order to verify the law of conservation of matter and energy in the diffusivity analysis, the area under the diffusivity curves was analyzed.

Figure 6 shows the graphic representation of the diffusivity behavior of the samples in the quartz cuvettes. It can be noticed that the evolution of the concentration of nanostructures in ethanol and in acetone is different. The results indicate that the particles can be easily dispersed in acetone suspensions. Diffusivity concentrations are indicative of fluid stability.

**Figure 7:**
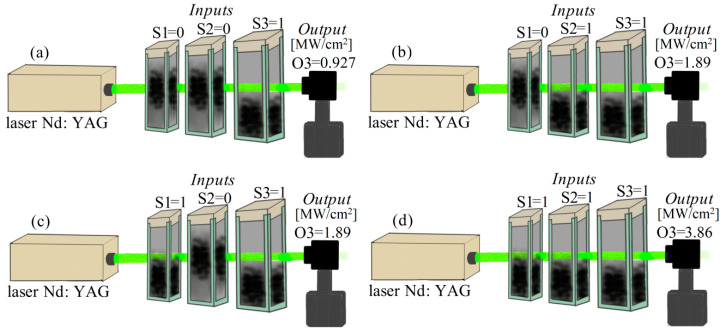
Experimental setup of the probabilistic gate in an AND configuration controlled by the particle position probabilities. The laser beam energy is absorbed by the CNTs position. (**a**) S1 = 0, S2 = 0; S3 = 1, O3 = 1 with 0.927 MW/cm^2^, (**b**) S1 = 0, S2 = 1; S3 = 1, O3 = 1 with 1.89 MW/cm^2^, (**c**) S1 = 1, S2 = 0; S3 = 1, O3 = 1 with 1.89 MW/cm^2^ (**d**) S1 = 1, S2 = 1; S3 = 1, O3 = 0 with 3.86 MW/cm^2^.

Regarding the experimental results, we consider that a probabilistic bit can be referred to an overlap of values associated with our samples. It was considered the value of the bits as the solute concentration suspended in the liquid samples. The logic states of the input’s gate were defined by the probability of the position of the particles in the fluid tested by an optical signal. The values in the measurement of the gate outputs and the experimental setup gate are shown in Figure 7.

The logic states of the output were designated by the irradiance of the laser beam as shown in Figure 7. The logical value 1 was assigned to an optical irradiance lower than 2 MW/cm^2^. Otherwise, the logical value is 0.

In addition, in Figure 7 the separation of the samples explored in different containers is schematized; the potential of the logic device can be extended to analyze a single nanofluid with diverse regions or thermodynamic properties of multiple phases.

**Figure 8:**
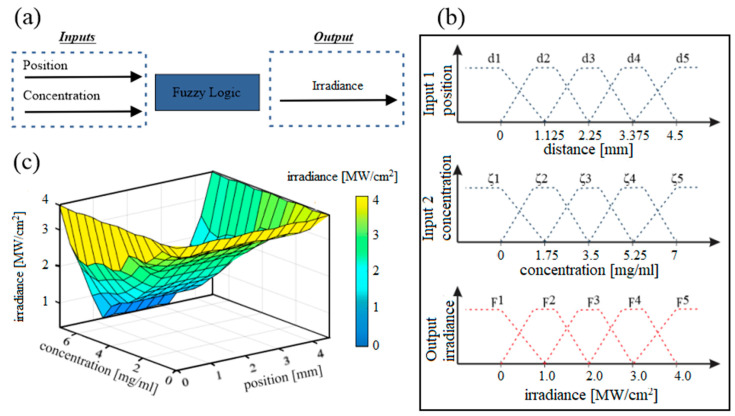
Graphic representation of fuzzy logic system. (**a**) diagram of system implemented; (**b**) parameter representation of the inputs and output variables for the fuzzy logic controller; (**c**) surface diagram of the fuzzy logic controller results for the nanofluid samples.

A probabilistic bit simulation was performed by using the fuzzy set theory. Figure 8a shows the fuzzy interference process to obtain the output value of the beam energy due to the concentration and position of the particles suspended in the nanofluid.

The fuzzy logic process was structured by using rules to define the behavior of nanofluids. The rules were established by the input variables distance, concentration, and the output energy. The sets defined by the domain of concentration x were represented by ζ1, ζ2, ζ3, ζ4, ζ5. Over domain y, the position are d1, d2, d3, d4, d5, and the output over domain z are ϝ1, ϝ2, ϝ3, ϝ4, ϝ5. The values of the domains represent the lowest, low, middle, high and higher level. Figure 8b shows a range of magnitudes of the domains in the fuzzy variables. For the fuzzification step, the peak values of the inputs were defined and the degree of belonging of the input variables to the associated fuzzy sets was determined. In this way, each input is fuzzified over all the belonging functions used in fuzzy rules.

For the evaluation of the fuzzy rules, the AND operator was used by considering the generalized form of the T-norm intersection to obtain a belonging function ϸ, which is defined as follows:

(9) $$ϸϘ\cap^{\;}Ϛ\left(x\right)=\;T\left(ϸϘ\left(x\right),\;ϸϚ\left(x\right)\right)$$

where Ϙ and Ϛ are fuzzy sets and x is the set variable.

In order to obtain the resulting values of the fuzzy logical system, the fuzzy set previously obtained is taken as an input to give an output value. The fuzzy controller receives the input data corresponding to the variables x_0_ and y_0_ by the Manmdani inference rules defined by:

(10) $$\begin{array}{c} ϸ{\zeta^{\prime }}_{1}\left(z\right)=ϸ{Ϙ}_{1}\left(x_{0}\right)\cap^{\;}ϸ{Ϛ}_{1}\left(y_{0}\right)\cap^{\;}ϸ\zeta_{1}\left(z\right)\\ ϸ{\zeta^{\prime }}_{2}\left(z\right)=ϸ{Ϙ}_{2}\left(x_{0}\right)\cap^{\;}ϸ{Ϛ}_{2}\left(y_{0}\right)\cap^{\;}ϸ\zeta_{2}\left(z\right)\\ \vdots \\ ϸ\zeta \left(z\right)=ϸ{\zeta ’}_{1}\left(z\right)\cup^{\;}\;ϸ{\zeta^{\prime }}_{2}\left(z\right)\cup^{\;}\ldots .ϸ{\zeta ’}_{n}\left(z\right) \end{array}$$

The belonging function values related to the input variables position and concentration are fuzzified. The minimum value between the belonging value of the input variables is calculated, in order to define the output set at the height of the smallest fuzzification value. The minimum belonging value of each rule is defined in order to limit the output set related to the energy of the beam. As a result, dimension sets are joined to calculate the membership function of the output set. The defuzzification of the sets is obtained by the following:

(11) $$\mathrm{Defuzzification}=\frac{\sum_{x=Ϙ}^{Ϛ}ϸϘ\left(x\right)x}{\sum_{x=Ϙ}^{Ϛ}ϸϘ\left(x\right)}$$

Figure 8c shows a superficial plot representing the fuzzy logic applied to the probabilistic bit system. The effect of the predictive diffuse system based on the behavior of the NPs suspended in the nanofluids and their impact on the optical irradiance of the system can be observed.

The results shown in Figure 8c demonstrate the effect of the input variables in the optical transmittance of the nanofluid. The relationship between the concentration and position of the NPs is also observed. The result of optical transmittance output shows that the maximum value mostly depends on the minimum concentration of the nanofluids which, at the same time, depends tightly on the diffusivity of the fluid.

**Figure 9:**
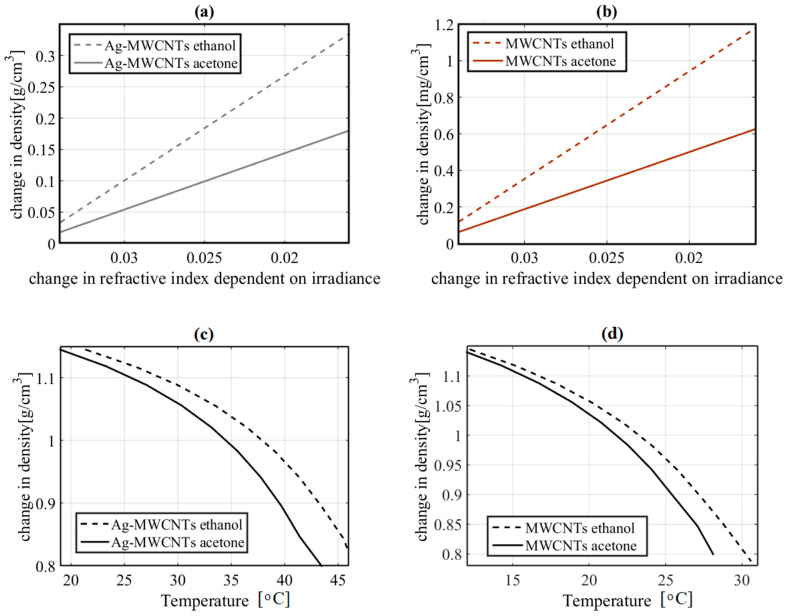
Numerical results of the induced change in density estimated by considering the change in refractive index in the nanofluids. (**a**) Ag-MWCNT samples, (**b**) MWCNT samples. Numerical results of the induced change in temperature estimated by the change in density in the nanofluids. (**c**) Ag-MWCNT samples, (**d**) MWCNT samples.

**Figure 10:**
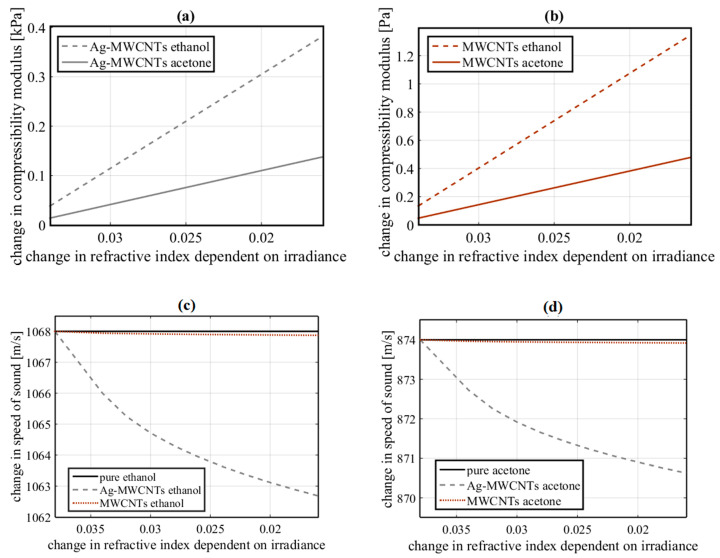
Numerical results of the induced change in compressibility modulus estimated by considering the experimental optical Kerr effect (OKE) in the nanofluids. (**a**) Ag-MWCNT samples, (**b**) MWCNT samples. (**c**) Change in the speed of sound due to Ag-MWCNT suspensions. (**d**) Change in the speed of sound due to MWCNT suspensions.

The optical Kerr effect, described by the magnitude of *n*_2_, can be associated with different physical mechanisms responsible for the third-order optical nonlinearity in a sample. In particular, molecular orientation, electronic polarization, electrostriction, magneto-optical phenomena or a thermal transfer can be related to a nonlinearity of the index [29]. In this aspect, a photothermal effect can be described as an optical Kerr effect that may be responsible for a change in density, as expressed by Equation (1). The change in the refractive index illustrated in Figure 9 and Figure 10 is the result of a change in the concentration of the samples. By using Equation (1), the change in refractive index was estimated at concentrations between 10 and 100% w of Ag-MWCNTs. It is notable that the refractive index can be related to the density of the samples. The numerical data resulting from Equation (1) confirm that the optical nonlinearities generated by TWM are significant in the changes in the mechanical properties of the nanofluids. The behavior for the change in density of the samples with Ag-MWCNTs ethanol and acetone nanofluids was analyzed and the results are shown in Figure 9a,b.

If the parameters described by the Lorentz–Lorenz relation are considered, it can be concluded that modification of the nanofluid density by dependence on optical irradiance must be present under high-optical irradiation. Figure 9c,d also shows the relation in temperature of the nanofluids considering the change in density.

Figure 10a,b shows the change in the compressibility modulus of the samples considering Equation (2). The changes in mechanical properties in the samples described by the Lorentz–Lorenz equation are caused by an induced nonlinear refractive index with dependence on optical irradiance and concentration of the samples. In the same way, an important contribution of different parameters such as the molecular weight of the chemical elements and the mean molecular polarizability of the samples ought to be considered.

Moreover, an approximation for the speed of sound through the Ag-MWCNTs nanofluids can be obtained by the compressibility modulus, and change in density. From the above, the numerical calculations require the calculus of density corresponding to elements that compose the sample. Density calculation was performed for suspensions with 2 mL of pure solvent, 0.0057 g to MWCNTs and 0.00246 g of metallic NPs. The previous weights correspond to 0.0082 g of Ag-MWCNTs with 30% of Ag NPs.

Figure 10 shows the change in the speed of sound of ethanol and acetone Ag-MWCNTs nanofluids. From Figure 10c, it is possible to observe the change in the speed of sound due to ethanol and acetone suspensions with MWCNTs without decorations. The result demonstrates that the addition of Ag-MWCNTs in nanofluids generates a higher change in the speed of sound of pure organic fluids. So, the refractive index of the Ag NPs adhered to the MWCNTs defines strong differences in the mechanical behavior of nanofluids according to the optical nonlinearities of the samples. The control of the mechanooptic properties exhibited by the studied samples gives an advantage in the field of nanoantenas. If the density of the involved nanofluids is very low, the phenomenon could not be observable due to the absence of enough NPs in their vicinity of the nanoantenas. On the other hand, if the density is very high, it is possible that high-density agglomerations will appear, preventing the reaction of the electromagnetic field of the nanoantenas. Additionally, the excitation beam could be absorbed together with an induced nonlinear refractive index depending on the optical irradiance and the concentration of the samples. Consequently, optical nonlinearities find important areas of application in the field of nanoantenas, and the dynamic behavior of NPs can control discrete variables associated with the physical-chemical changes of nanofluids.

The local control of the temperature of plasmonic nanoantenas has been studied and good results have been obtained for Ag NPs; however, the operating temperatures for hybrid materials present different advantages compared to pure Ag NPs [48]. In Figure 9b,c, the modified temperature operating range due to density and optical irradiance dependency is shown. The dissipation of energy within the metallic NPs is an important factor in the performance of nanoantenas [49]. In this work, the concentration gradients with respect to time in the fluid indicate the dynamic stability of the proposed nanofluids. Due to the nature of the diffusivity presented in the samples studied, the design of UV-VIS nanoantenas with minimal energy dissipation can be contemplated in comparison to sensitive nanoantenas with infrared response [50]. In addition to good light absorption and energy dissipation, plasmonic NPs have been studied for applications related to sensors and nanoswitches by coupling light with the NPs [51]. The application of optical nanoantenas in binary logic operations has also been explored [52]. In this work, the advantages of the plasmonic properties of metallic NPs have been considered as well as the optomechanical properties exhibited by MWCNTs. Using a fuzzy logic system, it was possible to design an AND logic gate with probabilistic signals. The system was structured by rules defined by the behavior resulting from the diffusivity simulations, and the optical response exhibited by the studied nanofluids. The operating principles depend on controlled sets of NPs with not only specific optical and mechanical properties, but also with quantum properties.

## 4. Conclusions

This work highlights the importance of metal/carbon nanostructures to control probabilistic functions assisted by dynamic and nonlinear optical properties. Nanoantenas in a fluid featuring a probabilistic NAND logic gate were proposed by the assistance of mechanical and nonlinear optical properties. The evolution of mechano-optical signals in simple organic liquids was modulated by the incorporation of Ag decorated MWCNTs. The participation of Ag NPs incorporated in MWCNTs seems to cause strong changes in off-resonance nonlinear optical interactions. The plasmonic properties of the metal nanoparticles allow for increasing the emission and control in the optical response due to the excitation of the electrons of the metal structures. Optically induced variations in density, compressibility modulus and speed of sound in metal/carbon-based nanofluids were analyzed considering a dynamic OKE. The mechanical and optical properties exhibited by MWCNTs show advantages over other nanostructured materials with the same dimensions. Due to the morphology of the CNTs, it is possible to consider nanoantenas for wireless communication in GHz and THz. CNTs allow developments in quantum and molecular communications due to their high rates of electrical and thermal conductivity and mechanical moldability. The plasmonic behavior derived from the decoration of the MWCNTs can also function as an oscillator for frequency modulation. This work can be a base for future research related to dynamic nonlinear optical systems assisted by hybrid nanostructures.

## Figures and Tables

**Table 1:** Truth table related to three probabilistic bits.

Inputs			Outputs		
S1	S2	S3	O1	O2	O3
0	0	0	0	0	0
0	0	1	0	0	1
0	1	0	0	1	0
0	1	1	0	1	1
1	0	0	1	0	0
1	0	1	1	0	1
1	1	0	1	1	1
1	1	1	1	1	0

## Supplementary Materials

The following are available online at https://www.mdpi.com/2079-4991/10/9/1886/s1, Supplementary material.
