# Supplementary material for: Navigation of Silver/Carbon Nanoantennas in Organic Fluids Explored by a Two-Wave Mixing
Source: Nanomaterials (Basel). 2020 Sep 21;10(9):1886. doi: 10.3390/nano10091886 (PMC7557386; doi:10.3390/nano10091886)
Supplement: Supplementary file 1 [file nanomaterials-10-01886-s001.pdf]

nanomaterials

Supplementary material

The electric fields in propagation through the samples can be described by considering the wave equation [29]:

$$\nabla^2 E_{\pm} = -\frac{n_{\pm}^2 \omega^2}{c^2} E_{\pm} \tag{S1}$$

In this mathematical equation E_+ and E_- represent the circular components of the left and right electric fields in the TWM interaction. The optical frequency is described by ω, c corresponds to the speed of the light and n is the refractive index dependent on irradiance which can be also described by [29]:

$$n_{\pm}^2 = n_0^2 + 4\pi\left(\chi_{1122}^{(3)}\left|E_{\pm}\right|^2 + (\chi_{1122}^{(3)} + \chi_{1212}^{(3)})\left|E_{\mp}\right|^2\right) \tag{S2}$$

where n_0 is the weak-field refractive index. $\chi_{1122}^{(3)}$ and $\chi_{1212}^{(3)}$ are the independent components of the third-order susceptibility tensor $\chi^{(3)}$.

In the interaction of a beam of light with nonlinear optical media can be involved an intensity-dependent refractive index n:

$$n = n_0 + n_2 I \tag{S3}$$

where, n_0 is the weak-field refractive index, n_2 in the nonlinear refractive index, and I refers to the intensity of the optical field.

The Lorentz–Lorenz equation relates the refractive index of a material to its polarizability α, and it can be written in terms of mass density ρ_m [29]:

$$\frac{n^2 - 1}{n^2 + 2} = \frac{4\pi}{3}\frac{N_A \rho m}{M}\alpha \tag{S4}$$

where N_A is the Avogadro's number and M is the molecular weight of the chemical element.
